# Patient risk factors and adverse drug interactions in the treatment of acute gouty arthritis in the elderly: a case report

**DOI:** 10.1186/1757-1626-2-6602

**Published:** 2009-04-28

**Authors:** Bernhard Zagler, Angelika Kaneppele, Peter Pattis, Ingrid Stockner, Günther Sitzmann, Josef Widmann, Heinrich Pernthaler, Christian J Wiedermann

**Affiliations:** 1 Division of Internal Medicine, Department of Medicine, Central Hospital of BolzanoL. Böhler Street 5, I-39100 Bolzano (BZ)Italy; 2 Division of Surgery I, Department of Surgery, Central Hospital of BolzanoL. Böhler Street 5, I-39100 Bolzano (BZ)Italy

## Abstract

**Introduction:**

Non-steroidal anti-inflammatory drugs and corticosteroids are both used in the treatment acute gouty arthritis and may adversely interact with colchicine. Gastrointestinal toxicity of colchicine is dose-dependent and can be aggravated by drug-drug and drug-patient interactions.

**Case presentation:**

Colonic perforation associated with second-line administration of colchicine for acute gouty arthrtitis was identified in an elderly man with several comorbidities who was also treated with non-steroidal anti-inflammatory drugs and corticostroids. Underlying diverticular disease was discovered at the time of surgical therapy.

**Conclusions:**

Initial treatment of acute gouty arthritis with non-steroidal anti-inflammatory drugs or corticosteroids may increase colchicine toxicity by subsequent pharmacokinetic and pharmacodynamic interaction in the gut wall. The literature is reviewed suggesting that diverticular disease should be included in the list of adverse event risk factors when colchicine is being considered.

## Case Presentation

A 74-year-old Caucasian male from Italy with chronic renal failure, ischemic heart disease, congestive heart failure, hypertension, diabetes mellitus and a history of gout presented to the emergency department with acute left-sided knee pain due to contusion after a recent fall. Other joints of the left extremity were not affected. After exclusion of acute hemarthrosis, internal knee derangements and musculotendinous strains by physical examination and radiograph, he was discharged home with the knee immobilized with an elastic wrap and crutches for pain associated with weight bearing. Three days later, severe pain, redness, swelling, and disability developed in the ankle of the same leg. Signs of inflammation extended beyond the confines of the ankle. Acute arthritis was suspected and, after exclusion of venous thrombosis by Doppler ultrasound, the patient was admitted for diagnostic work-up and treatment. His regular medications included daily oral carvedilol 18.75 mg, simvastatin 20 mg, ramipril 5 mg, amiodarone 200 mg, metformin 750 mg, omeprazole 20 mg, furosemide 125 mg, and warfarin 2.5 mg. The patient denied using herbal or over the counter products. He was independent in activities of daily living. There was no history of abdominal trauma.

Investigations before final diagnosis included blood and urine tests that showed elevated uric acid of 12.9 mg/dl (normal range, 2.5-8.0 mg/dl), mild leukocytosis, elevated erythrocyte sedimentation rate and C-reactive protein, hyperglycemia, and a reduced glomerular filtration rate of 34 ml/min; blood cultures were negative, liver function tests, amylase and serial troponins were normal. Knee radiographs excluded signs of stress fracture or traumatic process on bone or joint. A chest radiograph demonstrated signs of congestive heart failure; serial electrocardiograms confirmed atrial fibrillation but did not show signs of myocardial ischemia. Gouty ankle arthritis was diagnosed without joint aspiration; 100 mg diclofenac were administered for pain relief, metformin was stopped and normal saline was infused because of preexisting stage 3 moderate renal insufficiency. With no beneficial effect after 24 hours, the patient received second-line therapy with 1 mg colchicine orally. However, because of gastrointestinal toxicity, colchicine was stopped after the first dose. Instead, oral prednisone 40 mg/day was administered for three days and was effective.

Transient oliguric acute renal failure observed during the course of steroid treatment was interpreted as a side effect of NSAID aggravated by colchicine (rise in creatinine value from 2.4 to 5.2 mg/dL; normal range 0.5-1.4. mg/dL). During resolution of acute renal failure over the following four days, atypical chest and upper abdominal pain developed with worsening leukocytosis (rise of white blood cell count from 10,600/µL to 15,400/µL mg/dL; normal range 4,800-10,800/µL). With abdominal ultrasound showing a small amount of ascites and finally free air visible in abdominal radiograph, colonic perforation was diagnosed. Underlying diverticular disease was discovered at the time of surgical therapy consisting of resection and temporary colostomy. The postoperative clinical course was uneventful with no infectious complications other than peritoneal irritation. Allopurinol at a dose of 300 mg daily was prescribed for symptomatic hyperuricemia at 6 weeks after completion of surgical treatment.

## Discussion

NSAIDs and corticosteroids are both used in the treatment acute gouty arthritis and may adversely interact with colchicine. Gastrointestinal toxicity of colchicine is dose-dependent and can be aggravated by drug-drug and drug-patient interactions. The risk of colonic perforation may relate to intracolonic pressure and mucosal barrier function in the wall of diverticula [1]. The administration of NSAIDs, opioids, and corticosteroids for the treatment of arthritis is associated with an increased risk of colonic diverticular perforation [2]. NSAIDs and corticosteroids are indicated for arthritis; these two classes of drugs as well as colchicine are frequently used to treat and prevent recurrence of acute gout [3]. The maximum dose of colchicine for treating an acute attack of gout should be 6 mg because of its narrow therapeutic window, with dose-limiting gastrointestinal side-effects such as diarrhoea and vomiting [4]. Colchicine toxicity relates to its cellular anti-mitotic action and preferentially affects tissues that have a rapid turnover including the gastrointestinal tract [5]. As colchicine increases intracolonic pressure and impairs mucosal barrier functions [6], it may increase the risk of developing perforation. Colonic perforation in asymptomatic diverticulosis related to colchicine has not yet been reported.

Colchicine, long used to treat gout, arrests microtubule assembly and inhibits many cellular functions [7]. In addition to its use in gout, it has also been recommended in preventing attacks of familial Mediterranean fever, and in the treatment of primary biliary cirrhosis, amyloidosis, and condyloma acuminata [8,10]. At micromolar concentrations, it suppresses monosodium urate crystal-induced NACHT-LRR-PYD-containing protein-3 (NALP3) inflammasome-driven caspase-1 activation, IL-1beta processing and release, and L-selectin expression on neutrophils; at nanomolar concentrations, colchicine blocks the release of a crystal-derived chemotactic factor from neutrophil lysosomes, blocks neutrophil adhesion to endothelium by modulating the distribution of adhesion molecules on the endothelial cells, and inhibits monosodium urate crystal-induced production of superoxide anions from neutrophils [7]. Trial evidence supports its efficacy in acute gout and in preventing gout flares, but it has narrow therapeutic index, and overdosage is associated with gastrointestinal, hepatic, renal, neuromuscular, and cerebral toxicity, bone marrow damage, and high mortality [11].

Colchicine, which is increasingly considered third-line therapy only after NSAIDs and corticosteroids in patients at risk according to current guidelines, should be taken at an initial dose of 1 mg followed by 1 tablet every 2 hours until the gouty pain is relieved, gastrointestinal symptoms develop, or the maximum dose is reached; in elderly patients, those who weigh less than 50 kg and those with co-existing renal or hepatic disease, alternative therapy should be used or a maximum dose should not exceed 3 mg colchicine [12]. This dosage advice for colchicine has been amended recently, coinciding with the introduction of a 0.5 mg strength tablet. For otherwise healthy adults, the dosing interval has been increased from 2 hours to 6 hours, with a maximum dose of 2.5 mg in the first 24 hours and a maximum cumulative dose of 6 mg over four days. In elderly patients, patients with renal or hepatic impairment, and patients weighing less than 50 kg, other treatments should be considered or lower doses of colchicine used [12]. Patients should be warned of the symptoms of colchicine toxicity and advised to discontinue therapy immediately if they occur [3].

In our patient, low-dose colchicine was started as second line therapy and with the oral administration of a 1 mg tablet because 0.5-mg tablets are not yet available in Italy. Various factors may then have additionally modified toxicity of colchicine. (a) Colchicine is rapidly absorbed from the gastrointestinal tract after ingestion. It undergoes significant first-pass hepatic metabolism, which primarily involves deacetylation. Subsequent to this, the metabolites undergo widespread enterohepatic recirculation before being excreted in bile and faeces [7]. It is thought that the extended time period during which the gastrointestinal mucosal cells are exposed to colchicine may explain the prominence of the gastrointestinal symptoms of toxicity. (b) Renal clearance also accounts for 10-20% of colchicine removal and if renal function is normal, larger fractions can be excreted via this route if a toxic amount has been ingested. Moderate renal dysfunction may, thus, have increased enterohepatic colchicine levels [12]. (c) Increased urinary excretion also occurs in the presence of hepatic disease, as there is a reduction in the capacity for deacetylation; and, if renal and hepatic diseases coexist, the possibility of toxicity greatly increases [13]. Hepatic function was normal in our patient making it unlikely that such a mechanism contributed to increased toxicity. (d) Cytochrome P450 3A4 and the drugs that bind to it influence colchicine's pharmacokinetics and pharmacodynamics; CYP3A4 inhibitors may increase the levels/effects of colchicine. Diclofenac administered as first-line treatment for the patient's acute gout and, more importantly, amiodarone which the patient had taken chronically inhibit CYP3A4 [14] and, therefore, may have augmented toxicity. (e) The multidrug transporter P-glycoprotein, and drugs that bind to it, may also affect colchicine pharmacology: P-glycoprotein is an ATP-dependent phospho-glycoprotein that is located in the cell membrane and interacts with various drugs. When binding drugs are prescribed in combination, changes in P-glycoprotein activity may lead to intracellular accumulation of colchicine; this list of drugs includes prednisolone [15] which was administered as third-line therapy of acute gout. A combination of several pharmacokinetic and pharmacodynamic factors therefore may well have increased the gastrointestinal toxicity of colchicine.

The adverse gastrointestinal event of colonic perforation likely relates to previously undiagnosed diverticular disease. In the Naranjo Adverse Drug Reaction Probability Scale [16], a score of +3 indicates the overall probability that the adverse event represents an adverse reaction to colchicine as “possible” (Table 1). Drug-Drug and drug-patient interaction likely have contributed to the treatment's complication. As intracolonic pressure plays a prominent role in macroscopic perforation of colonic diverticular disease [2], its augmentation during diarrhea of colchicine's gastrointestinal toxicity may give this drug a predominant causative role.

**Table 1:** Naranjo ADR Probability Scale. The use of the scale involves answering a series of questions about the adverse event, and then calculating a final score that provides some indication of the overall probability that the adverse event represents an adverse reaction to a drug

1	Are there previous conclusive reports on this reaction?	
	Yes (+1) No (0) Don't know (0)	+1*
2	Did the adverse event appear after the suspected drug was administered?	
	Yes (+2) No (−1) Don't know (0)	+2
3	Did the adverse reaction improve when the drug was discontinued, or a specific antagonist was administered?	
	Yes (+1) No (0) Don't know (0)	0
4	Did the adverse reaction reappear when the drug was readministered?	
	Yes (+2) No (−1) Don't know (0)	0
5	Are there alternative causes (other than the drug) that could on their own have caused the reaction?	
	Yes (−1) No (+2) Don't know (0)	−1
6	Did the reaction reappear when a placebo was given?	
	Yes (−1) No (+1) Don't know (0)	0
7	Was the drug detected in the blood (or other fluids) in concentrations known to be toxic?	
	Yes (+1) No (0) Don't know (0)	0
8	Was the reaction more severe when the dose increased, or less severe when dose was decreased?	
	Yes (+1) No (0) Don't know (0)	0
9	Did the patient have a similar reaction to the same or similar drug in any previous exposure?	
	Yes (+1) No (0) Don't know (0)	0
10	Was the adverse event confirmed by any objective evidence?	
	Yes (+1) No (0) Don't know (0)	+1
	Total	+3

The final score allows some basis for an objective assessment of the likelihood that an ADR may have occurred:

> 9 = highly probable.

> 5 − 8 = probable.

> 1 − 4 = possible.

≤ 0 = doubtful.

*Answers given for the patient reported.

In conclusion, drug treatment of acute gouty arthritis with NSAID and corticosteroids may have increased colchicine toxicity by pharmacokinetic and pharmacodynamic interaction in the wall of diverticula. Increased intracolonic pressure of colchicine-induced diarrhoea was possibly contributing to the patient's colonic perforation. In patients with risk factors for colonic perforation including age, renal insufficiency, NSAIDs and corticosteroids colchicine should best be avoided. Patients receiving therapy with colchicine, especially those with renal comorbidities and concomitant drug treatment, should be carefully monitored for complications of colchicine's gastrointestinal side effects. Oral colchicine formulations with 0.5-mg tablets should be made available more widely. Diverticular disease should be included in the list of adverse event risk factors when therapy with colchicine is under consideration. Colchicine possibly is associated with an increased risk of colonic diverticular perforation.

## List of abbreviations

ATP: Adenosine triphosphate
NALP3: NACHT-LRR-PYD-containing protein-3
NSAIDs: Non-steroidal anti-inflammatory drugs

## References

[bib-001] Morris CRHI, Stebbings WSL, Speakman CTM, Kennedy HJ, Hart AR (2002). Epidemiology of perforated colonic diverticular disease. Postgrad Med J.

[bib-002] Piekarek K, Israelsson LA (2008). Perforated colonic diverticular disease: the importance of NSAIDs, opioids, corticosteroids, and calcium channel blockers. Int J Colorectal Dis.

[bib-003] Zhang W, Doherty M, Bardin T, Pascual E, Barskova V, Conaghan P (2006). EULAR Standing Committee for International Clinical Studies Including Therapeutics. EULAR evidence based recommendations for gout. Part II: Management. Report of a task force of the EULAR Standing Committee for International Clinical Studies Including Therapeutics (ESCISIT). Ann Rheum Dis.

[bib-004] Morris I, Varughese G, Mattingly P (2003). Colchicine in acute gout. BMJ.

[bib-005] Stemmermann GM, Hayashi T (1972). Colchicine intoxication: a reappraisal of its pathology, based on a study of three fatal cases. Hum Pathol.

[bib-006] Torbenson M, Montgomery EA, Iacobuzio-Donahue C, Yardley JH, Wu TT, Abraham SC (2002). Colchicine effect in a colonic hyperplastic polyp. A lesion mimicking serrated adenoma. Arch Pathol Lab Med.

[bib-007] Nuki G (2008). Colchicine: its mechanism of action and efficacy in crystal-induced inflammation. Curr Rheumatol Rep.

[bib-008] Zemer D, Revach M, Pras M, Modan B, Schor S, Sohar E, Gafni J (1974). A controlled trial of colchicine in preventing attacks of familial mediterranean fever. N Engl J Med.

[bib-009] Oo YH, Neuberger J (2004). Options for treatment of primary biliary cirrhosis. Drugs.

[bib-010] Cohen AS, Rubinow A, Anderson JJ, Skinner M, Mason JH, Libbey C, Kayne H (1987). Survival of patients with primary (AL) amyloidosis. Colchicine-treated cases from 1976 to 1983 compared with cases seen in previous years (1961 to 1973). Am J Med.

[bib-011] Terkeltaub RA (2008). Colchicine Update: 2008. Semin Arthritis Rheum.

[bib-012] Hood RL (1994). Colchicine poisoning. J Emerg Med.

[bib-013] Borron SW, Scherrmann JM, Baud FJ (1996). Markedly altered colchicine kinetics in a fatal intoxication: Examination of contributing factors. Hum Exp Toxicol.

[bib-014] Tang W (2003). The metabolism of diclofenac--enzymology and toxicology perspectives. Curr Drug Metab.

[bib-015] Niel E, Scherrmann JM (2006). Colchicine today. Joint Bone Spine.

[bib-016] Naranjo CA, Busto U, Sellers EM, Sandor P, Ruiz I, Roberts EA (1981). A method for estimating the probability of adverse drug reactions. Clin Pharmacol Ther.

